# A curriculum-integrated learning experience linking experimental pharmacology, cell culture and Nrf2-related gene expression analysis in undergraduate biotechnology education

**DOI:** 10.3389/fphar.2026.1889438

**Published:** 2026-06-29

**Authors:** Cristina García-Bonillo, Sandra Atienzar-Aroca, Martín Pérez-Leal

**Affiliations:** Department of Biosciences, Faculty of Health Sciences, Universidad Europea de Valencia, Valencia, Spain

**Keywords:** curriculum integration, experiential learning, experimental pharmacology, gene expression analysis, undergraduate biotechnology education

## Abstract

**Background:**

Undergraduate biotechnology education requires integrated, experiential approaches that help students connect pharmacological modulation, cellular models, molecular analysis and data interpretation within coherent biological problems. This study describes and evaluates a curriculum-integrated learning experience linking experimental pharmacology, cell culture and Nrf2-related gene expression analysis in third-year Biotechnology students.

**Methods:**

A total of 32 students participated in the survey-based evaluation. The activity used A549 cells, sulforaphane as an Nrf2-activating compound and tert-butyl hydroperoxide as a pro-oxidant stimulus. Students followed an integrated workflow combining treatment design, cell culture handling, Nrf2-related gene selection, PCR-based gene expression analysis and scientific writing. Perceived learning was assessed using paired pre–post Likert-scale items and analyzed with the Wilcoxon matched-pairs signed-rank test. Post-intervention perceptions, satisfaction and open-ended feedback were analyzed descriptively.

**Results:**

Students showed significant improvements in all five pre–post items assessing perceived confidence or familiarity (*p value* < 0.0001). The proportion of students showing improvement ranged from 81.3% to 100%. Post-intervention responses indicated positive perceptions of interdisciplinary integration, technical competencies, digital competencies, data interpretation and research/professional development. Overall satisfaction was high, with a mean score of 8.50 ± 1.10 out of 10; 87.5% of students rated the activity with a score of 8 or higher. Qualitative feedback highlighted interdisciplinary integration and global understanding of the experimental workflow as key strengths.

**Conclusion:**

This curriculum-integrated learning experience was associated with perceived gains across pharmacological, cellular and molecular components of a biotechnology workflow. The intervention may provide a transferable model for integrating experiential, research-oriented and competency-based learning in undergraduate biotechnology education.

## 1 Introduction

Undergraduate biotechnology education requires learning experiences that help students connect experimental procedures, molecular mechanisms, data interpretation, and scientific communication within coherent biological problems. However, practical teaching in biomedical and life science degrees is frequently organized around discipline-specific laboratory sessions, which may limit students’ ability to understand how different experimental stages contribute to answering the same research question. Integrated curricula have been proposed as a strategy to promote meaningful connections across disciplines and to support deeper, transferable learning (Harden, 2000; Brauer and Ferguson, 2015). This is especially relevant in biotechnology, where professional and research practice commonly require the combined use of cell culture models, chemical or pharmacological modulation, molecular biology techniques, and data analysis.

Active and experiential learning approaches provide a suitable framework for this type of integration. Active learning improves student performance in science, technology, engineering and mathematics disciplines compared with traditional lecture-based instruction (Freeman et al., 2014), while experiential learning emphasizes authentic practice, reflection and application as central components of knowledge construction (Yardley et al., 2012). In experimental biosciences, this implies moving beyond the mechanical execution of protocols and helping students understand why each step is performed, how methodological decisions influence downstream results and how evidence is interpreted within a biological context.

Course-based undergraduate research experiences (CUREs) offer a scalable way to incorporate authentic research practices into undergraduate teaching. These experiences involve scientific practices, collaboration, interation and the generation or interpretation of data in a research-oriented context (Auchincloss et al., 2014; Bangera and Brownell, 2014). More recent CURE models have also emphasized the value of integrating bioinformatics, writing, metacognition and collaborative work into undergraduate biology education (Yang and Korsnack, 2024). This is particularly relevant for biotechnology students, since modern life science training increasingly requires digital and data-related competencies, including the use of biological databases, primer design tools, and gene expression analysis. Recent genomics education frameworks further support the need to incorporate genomics concepts into undergraduate curricula in ways that connect molecular information with biological function and experimental design (Reed et al., 2025).

Building on these principles, contemporary science education has increasingly adopted active, problem-based, case-based, and competency-oriented approaches. In pharmacology, a recent network meta-analysis involving more than 21,000 students suggested that team-based learning, problem-based learning combined with case-based learning and flipped classroom strategies may improve several educational outcomes (Xiao et al., 2023). Contemporary reviews also highlight the need to move from content-heavy, discipline-isolated teaching towards learner-centered and competency-based approaches (Fasinu and Wilborn, 2024), while recent initiatives have emphasized the value of contextualized resources that help students transfer pharmacological knowledge to applied training environments (Pérez-Leal et al., 2026). In biotechnology degrees, this transition is particularly meaningful when pharmacological reasoning is embedded within cellular and molecular systems. Accordingly, integrating pharmacology with cell culture and functional genomics can help students follow the experimental continuum from cellular model handling and treatment application to downstream gene expression analysis, supporting a more coherent understanding of biological modulation than isolated, discipline-specific laboratory activities.

Based on this rationale, we designed a curriculum-integrated learning experience linking cell culture, experimental pharmacology and Nrf2-related gene expression analysis in undergraduate biotechnology education. The activity used A549 cells exposed to tert-butyl hydroperoxide (tBHP) as a pro-oxidant stimulus and sulforaphane as an Nrf2-activating compound. Sulforaphane is widely used as a pharmacological/nutrigenomic activator of the Nrf2 pathway, promoting antioxidant and cytoprotective gene expression through modulation of KEAP1–Nrf2 signaling (Kensler et al., 2013; Houghton et al., 2016). This model allowed students to explore the transcriptional regulation of antioxidant and cytoprotective genes related to the Nrf2 pathway, a central cellular defense mechanism against oxidative stress (Loboda et al., 2016). This study aimed to evaluate the perceived educational impact of this integrated, experiential and research-oriented teaching intervention, with particular attention to students’ perceived learning, satisfaction and perception of interdisciplinary coherence.

## 2 Materials and methods

### 2.1 Study design and educational setting

This study was designed as a single-cohort educational intervention based on the descriptive analysis of students’ perceived learning, satisfaction and perception of interdisciplinary coherence. The intervention was implemented during the second semester of the third year of the Biotechnology degree at Universidad Europea de Valencia, Spain. A total of 32 undergraduate students participated in the activity and provided valid survey responses for analysis. Participants were third-year undergraduate students who had previously completed foundational coursework in biomedical and life science subjects, as well as basic laboratory practice, as part of their degree curriculum. The integrated activity was embedded within regular practical teaching activities, whereas participation in the anonymous survey-based evaluation was voluntary. Only students who provided consent for the use of their anonymized data for teaching innovation and research purposes were included in the analysis.

Three subjects were involved in the integrated experience: Cell Culture and Tissue Engineering, Pharmacology, and Functional Genomics and Transcriptomics. The activity was conceived as a curriculum-integrated learning experience in which students addressed a shared biological problem across coordinated teaching units, rather than completing independent and discipline-specific laboratory sessions. The educational design was based on experiential and project-oriented learning, with each subject contributing a specific component to a common experimental workflow.

**FIGURE 1:**
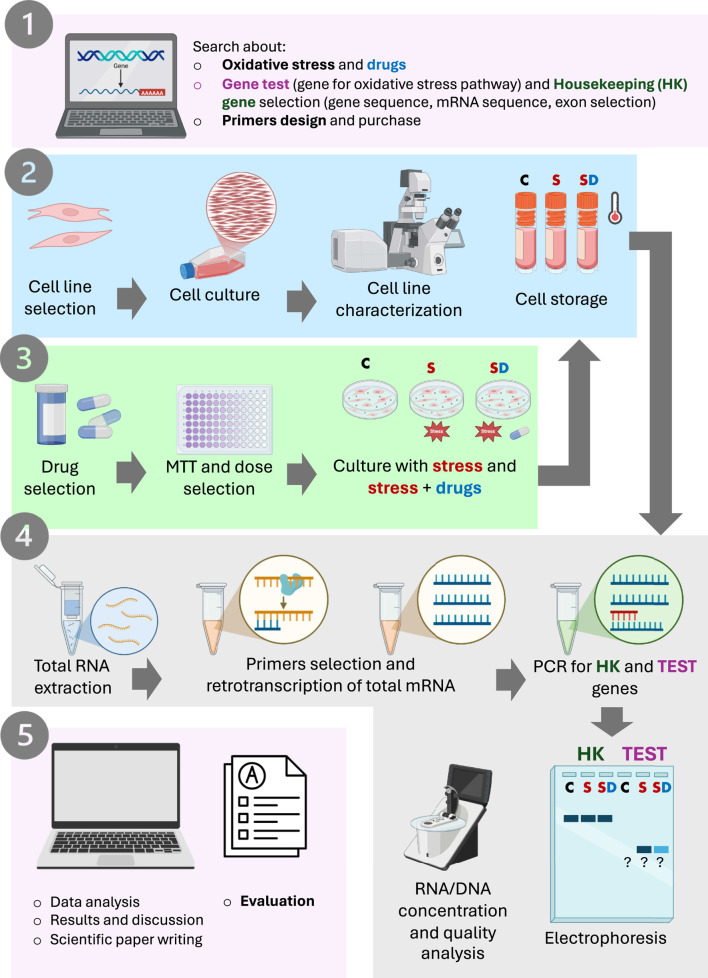
Schematic overview of the curriculum-integrated learning experience. Students followed a sequential workflow across three coordinated subjects, beginning with literature search, gene selection and primer design, followed by A549 cell culture, pharmacological modulation with sulforaphane, oxidative stimulation with tBHP, RNA extraction, cDNA synthesis, PCR-based gene expression analysis and scientific writing. The workflow illustrates the integration of cell culture, pharmacology, and functional genomics/transcriptomics into a shared research-oriented learning experience. Control culture (C), culture with oxidizing agent (S), and culture with oxidizing agent plus added drug (SD).

The overall structure of the intervention is summarized in Figure 1, which shows the sequential flow followed by students from cell culture handling and pharmacological treatment to molecular analysis, interpretation of results and scientific communication.

### 2.2 Description of the integrated learning intervention

The intervention was structured around an experimental model using A549 cells, sulforaphane as an Nrf2-activating compound and tert-butyl hydroperoxide (tBHP) as a pro-oxidant stimulus. This model was selected because it allowed students to connect cellular handling, pharmacological modulation and targeted gene expression analysis within a biologically coherent framework. The central educational question was whether sulforaphane-mediated activation of Nrf2 signaling could modulate the expression of antioxidant and cytoprotective genes in cells exposed to oxidative stress.

As shown in Figure 1, students followed a sequential workflow distributed across the three participating subjects. Students were organized into stable pairs that were maintained throughout the three participating subjects. Each pair completed the practical stages of the workflow and prepared the final scientific report, thereby promoting continuity across the integrated learning experience. Student autonomy was maintained throughout the intervention within a guided teaching framework. Instructors introduced the overall experimental rationale, safety considerations and methodological instructions at each stage, and remained available to answer questions or support troubleshooting during the sessions. However, the practical procedures were carried out by the students themselves, including cell culture monitoring, treatment application, primer-related decisions, protocol execution and interpretation of results.

In the Cell Culture component, students initiated the practical workflow from the initial handling of A549 cultures and maintained their own cell cultures throughout the project, thereby mimicking a real-world research workflow within a teaching setting. Students worked with A549 cells under sterile conditions and performed basic procedures related to cell maintenance, expansion, seeding, monitoring of confluence and morphology, medium handling and preparation of cell samples for subsequent experimental stages. This part of the intervention provided the cellular basis for the pharmacological and molecular analyses.

In the Pharmacology component, students focused on the design and application of the experimental treatments. They calculated concentrations and dilutions, prepared sulforaphane treatments and applied tBHP as an oxidative stimulus under controlled conditions. The activity emphasized the rationale of the experimental groups, including untreated control cells, cells exposed to oxidative stress and cells receiving pharmacological modulation with sulforaphane. This stage allowed students to connect concepts such as drug action, biological response, experimental controls and treatment-dependent effects.

In the Functional Genomics and Transcriptomics component, students completed the molecular stage of the integrated workflow. This included reviewing gene expression principles and oxidative stress pathways, exploring biological databases, selecting Nrf2-related target genes and reference/control genes, and designing a targeted gene expression strategy. In the laboratory, students extracted RNA, synthesized cDNA and performed PCR-based analysis followed by electrophoretic visualization of amplicons. This component allowed students to connect the previous cellular and pharmacological interventions with molecular readouts, reinforcing the relationship between experimental design, gene expression analysis and biological interpretation.

The final learning output was the preparation of a scientific report in article format. Through this task, students were expected to integrate the rationale of the experimental model, describe the methods used across the three subjects, interpret the results obtained and discuss the limitations of the experimental approach. This written output was included to promote scientific communication, critical analysis and integration of disciplinary knowledge.

### 2.3 Evaluation instruments

Students’ perceived learning and perceptions of the integrated activity were evaluated using anonymous questionnaires. The evaluation was mainly aligned with Kirkpatrick Level 1, focusing on students’ reaction and satisfaction with the learning experience, and was complemented by self-reported pre–post measures of perceived learning. Data collection included:

1. A pre-intervention questionnaire assessed students’ perceived familiarity and confidence with key concepts and techniques related to cell culture, oxidative stress, pharmacological treatment, RNA extraction, cDNA synthesis, PCR-based gene expression analysis and experimental design.
2. A post-intervention test measuring perceived gains in understanding of cell culture, oxidative-stress induction, pharmacological treatment, RNA extraction, cDNA synthesis, and PCR.
3. A final satisfaction survey combining Likert-scale items and open-ended questions to evaluate students’ perception of the usefulness, coherence and applicability of the integrated learning experience. Specific items addressed the perceived connection between the three subjects, the usefulness of the activity for understanding oxidative stress and Nrf2-related gene expression, and the perceived contribution of the experience to experimental, analytical, digital and research-related competencies.

The questionnaire was specifically designed for this teaching innovation project, as no previously validated instrument fully matched the integrated pharmacology, cell culture and gene expression workflow evaluated in the present study. Content and face validity were assessed by the teaching team, composed of instructors from the participating subjects, who reviewed the items for clarity, relevance and alignment with the intended learning objectives of the activity.

The complete questionnaire used for data collection is provided as Supplementary Material S1.

In addition, the scientific article prepared by students was considered as a final learning output aimed at promoting integration of the experimental rationale, methodological understanding, interpretation of results and scientific communication. This assignment was prepared in stable pairs and assessed as part of the ordinary academic evaluation of the participating subjects. Assessment focused on the clarity of the experimental rationale, methodological description, interpretation of results, discussion of limitations and integration of the three disciplinary components. Negative or inconclusive experimental results were not penalized when students appropriately identified possible methodological causes, discussed limitations and interpreted their findings critically.

Predefined feasibility and acceptability criteria were established by the teaching team before data analysis, based on the educational objectives of the intervention and previous teaching experience with practical laboratory-based activities. These criteria were not intended as externally validated benchmarks of effectiveness, but as pragmatic indicators to determine whether the integrated activity was sufficiently well received and educationally useful to support future implementation. They included: ≥80% of students rating overall satisfaction as 8/10 or higher, reflecting a target of high student acceptability; ≥70% of students showing perceived improvement in each pre–post item, reflecting the expectation that a clear majority of students would report gains across the evaluated domains; and the presence of positive qualitative comments regarding interdisciplinary integration and the educational value of the project.

### 2.4 Data analysis

Pre–post questionnaire items were analyzed using medians and interquartile ranges. Since responses were paired and based on 5-point Likert scales, pre- and post-intervention scores were compared using the Wilcoxon matched-pairs signed-rank test, with statistical significance set at *p* < 0.05. Individual response changes were also classified as improved, unchanged or decreased, and visualized using Sankey diagrams to represent the direction and distribution of students’ perceived learning gains.

Post-intervention Likert-scale items assessing interdisciplinary integration, technical competencies, digital competencies and research/professional development were analyzed descriptively using response distributions and percentages. Overall satisfaction was analyzed descriptively using mean, standard deviation, median, interquartile range and response distribution. Open-ended responses were reviewed thematically and grouped into recurrent categories. Exploratory internal consistency of the post-intervention Likert-scale domains was assessed using Cronbach’s α. Given the exploratory nature of the study and the limited number of items included in each domain, α values were interpreted cautiously as indicators of internal consistency rather than as definitive evidence of questionnaire reliability (Tavakol and Dennick, 2011).

### 2.5 Ethical considerations

The study was approved by the Research Committee of Universidad Europea under approval code 2026-755. Ethical approval was obtained prior to survey data collection. The data analyzed in this study were collected during the second semester of the 2025–2026 academic year, corresponding to 2026. The study used anonymized educational survey data collected within the context of a teaching innovation project. Participation in the survey-based evaluation was voluntary, and students were informed that their responses would be analyzed anonymously and would not affect their academic evaluation. Only responses from students who provided consent for the use of their anonymized data for teaching innovation and research purposes were included in the analysis.

## 3 Results

### 3.1 Student-generated experimental outputs from the integrated research workflow

Students conducted a literature search on oxidative stress, the Nrf2 pathway, and genes potentially involved in stress responses in cultured cells. They consulted major biological databases, including the National Center for Biotechnology Information (NCBI), Nucleotide Database, RefSeq (Reference Sequence Database), GenBank, and the National Institutes of Health (NIH) resources. Based on this exploration, students selected genes of interest and retrieved the corresponding FASTA sequences of putative mRNAs—including gene variants—from the NCBI mRNA Sequence Database. For each target mRNA, one exon was selected, and the ApE (Davis and Jorgensen, 2022). *ApE - A Plasmid Editor* (v3.1.9, 17 February 2026) (Software), utah.edu) software was used to generate forward and reverse primers (Table 1). Students repeated this workflow for the housekeeping (HK) gene of their choice. All primers were ordered without correction, allowing students to interpret and troubleshoot any potential design errors during the subsequent PCR experiments.

**TABLE 1:** Primer sequences designed by students for both housekeeping and target genes included in the targeted gene expression analysis. Housekeeping genes were selected for normalization purposes, while target genes represent key regulators of oxidative stress, redox homeostasis, and stress-response signaling. For each gene, students retrieved mRNA FASTA sequences from the NCBI mRNA Sequence Database, selected exon regions, and generated forward (F_) and reverse (R_) primers.

Gene	Function	Primer	Sequence 5′ 3′
HPRT1	Purine salvage enzyme; stable reference gene	F_HPRT1	AGT​TCT​GTG​GCC​ATC​TGC​TT
		R_HPRT1	AAC​AAT​CCG​CCC​AAA​GGG​AA
GAPDH	Glycolytic enzyme widely used as a reference gene	F_GAPDH	AGA​ACA​TCA​TCC​CTG​CCT​CTA​CTG
		R_GAPDH	AAA​GTG​GTC​GTT​GAG​GGC​AAT​G
G6PD	Key enzyme in the pentose phosphate pathway; maintains NADPH levels	F_G6PD	GACGACGAAGCGCAGACA
		R_G6PD	GCC​TTG​AAG​AAG​GGC​TCA​CT
HSPA1A	Heat-shock protein involved in protein folding and stress response	F_HSPA1A	GCC​GAG​AAG​GAC​GAG​TTT​GA
		R_HSPA1A	ACA​GCA​ATC​TTG​GAA​AGG​CCC
B2M	Component of MHC class I; common normalization gene	F_B2M	GCT​CGC​GCT​ACT​CTC​TCT​TT
		R_B2M	TCA​TCC​AAT​CCA​AAT​GCG​GC
GAPDH (variant 2)	Alternative transcript of GAPDH used for normalization	F_GAPDH_2	TGC​ACC​ACC​AAC​TGC​TTA​GC
		R_GAPDH_2	GGC​ATG​GAC​TGT​GGT​CAT​GAG
TBP	TATA-binding protein; core transcription factor	F_TBP	ATG​CCC​TTC​TGT​AAG​TGC​CC
		R_TBP	GAG​GTG​GAA​TGT​GTC​TGG​CA
ACTB	β-actin; cytoskeletal structural protein	F_ACTB	GCA​GGA​GTA​TGA​CGA​GTC​CG
		R_ACTB	TGT​GTG​GAC​TTG​GGA​GAG​GA
IPO8	Nuclear transport protein; stable housekeeping gene	F_IPO8	AAG​GGG​ATG​AAA​TGA​GGG​G
		R_IPO8	GAC​AAA​GGT​CAA​AGG​GGA​AAG
P27 (CDKN1B)	Cyclin-dependent kinase inhibitor; regulates cell-cycle arrest	F_P27	AAC​GTG​CGA​GTG​TCT​AAC​G
		R_P27	TTG​ACG​TCT​TCT​GAG​GCC​AGG
mTOR	Central regulator of cell growth, metabolism, and stress responses	F_mTOR	CTC​TGT​GCA​TCT​GCT​CAG​CC
		R_mTOR	GACCTCACCCCTGCCACC
HMOX1	Heme oxygenase-1; antioxidant enzyme induced by oxidative stress	F_HMOX1	TGT​CTC​AAA​CCT​CCA​AAA​GCC​C
		R_HMOX1	CCA​CAG​TGC​CGT​TAA​ACA​CCT​C
NFE2L2 (NRF2)	Master transcription factor controlling antioxidant responses	F_NFE2L2	GGG​GTA​AGA​ATA​AAG​TGG​CTG​CTC
		R_NFE2L2	TTG​CTG​CAG​GGA​GTA​TTC​ACT​AGG
PTEN	Tumor suppressor regulating PI3K/AKT signaling and redox balance	F_PTEN	ACA​TCC​TAC​CCC​TTT​GCA​C
		R_PTEN	TCC​CTC​CAT​TCC​CCT​AAC​C
NOX1	NADPH oxidase generating reactive oxygen species (ROS)	F_NOX1	CTG​TCC​AGA​GAA​GGA​AGG​CAG
		R_NOX1	CTG​CCT​TCC​TTC​TCT​GGA​CAG
GPX2	Glutathione peroxidase; detoxifies hydrogen peroxide	F_GPX2	TGA​ATG​GGC​AGA​ACG​AGC​AT
		R_GPX2	ATT​CTG​TGA​AGG​CCC​AGA​GC
PI3K	Kinase involved in survival and stress-response signaling	F_PI3K	CAG​ACG​ACA​GAT​GGA​CAG​TGT​G
		R_PI3K	CAA​TGG​TGT​GAC​CGA​AGA​CAG​G
LAM2 (laminin subunit)	Extracellular matrix protein linked to cell adhesion and stress adaptation	F_LAM2	GAC​GAC​AAC​TTC​CTT​GTG​CC
		R_LAM2	TGC​CTC​CCT​TCT​GAG​ATT​GC
NRF2	Antioxidant response regulator; controls cytoprotective gene expression	F_NRF2	TCC​TAC​TGT​GAT​GTG​AAA​TGC
		R_NRF2	TGA​TTC​AAC​ATA​CTG​ACA​CTC​C

In parallel, students evaluated the pharmacological conditions to be used for the analysis of gene expression changes in the selected targets. In the Pharmacology component, they performed cell viability assays with sulforaphane, generated dose–response curves and estimated the IC_50_ by fitting the data to a four-parameter sigmoidal model. Based on these results and on the experimental feasibility of the integrated workflow, a working concentration of 10^–5^ M sulforaphane was selected for subsequent treatment experiments. After selecting and ordering the primers for the test and housekeeping genes, students proceeded with the culture of A549 cells, during which they exercised a high degree of autonomy in managing the experimental procedures. The cultures were subsequently characterized through observation of cell morphology and growth progression. In this context, students independently determined the appropriate timing for passaging the cells upon reaching confluence.

Following stimulation with tBHP-induced oxidative stress and subsequent treatment with the selected drug, the cells were preserved in *RNAlater* (Sigma Aldrich®) for later transcriptomic analysis. During RNA extraction, cDNA synthesis, and PCR amplification, the students were required to select the appropriate RNA extraction protocol for their cell culture, based on the PureLink™ RNA Mini Kit (ThermoFisher®). They also had to choose the suitable primers for cDNA synthesis (either oligo (dT)18 primers (ThermoFisher®) or random hexamers (N8080127, ThermoFisher®)) as well as select the reagents necessary to prepare the PCR mix and the appropriate polymerase from the various SuperScript™ reverse transcriptase options provided. Subsequently, the students selected the reagents for the standard PCR mix, including different Taq polymerase options and potential additives. The students were responsible for designing the thermocycler programs for both reverse transcription and amplification, considering the different melting temperatures (Tm) of the primers they had designed. At each stage, they were required to assess RNA and cDNA concentration and quality using a spectrophotometer (Nanodrop) to normalize concentrations and prepare the PCR mixes appropriately. This also enabled them to identify potential issues in their protocols, such as contamination with organic compounds or alcohols, which several students encountered.

**FIGURE 2:**
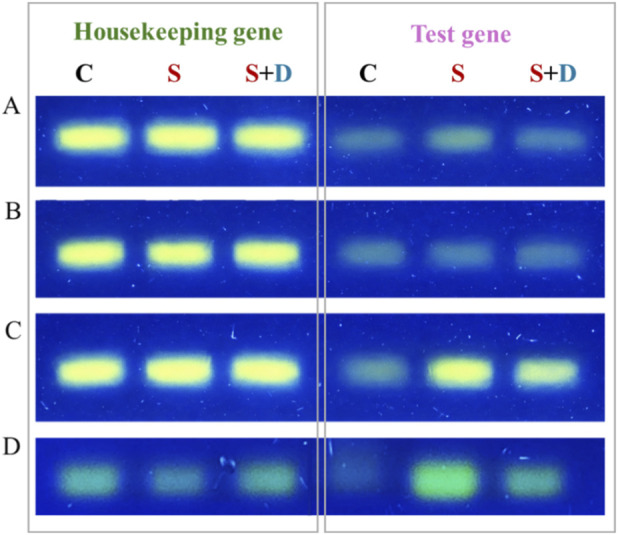
Representative electrophoresis gel showing the project results obtained by 4 different students **(A–D)**. The gel includes the three experimental conditions assessed: control cells (C), cells exposed to oxidative stress (S), and cells exposed to oxidative stress and treated with the drug (S + D). Samples were arranged so that the housekeeping gene selected by each student appears under the three conditions (left), while the corresponding test gene appears under the same three conditions (right). Panels **(A–D)** illustrate that the housekeeping gene is expressed at comparable levels across all four examples regardless of condition, whereas the test genes display clear differences in fluorescence intensity, consistent with condition-dependent changes in gene expression.

Gene expression changes were evaluated by electrophoresis (Figure 2), where variations in fluorescence intensity were interpreted as indicative of differences in PCR-amplified cDNA abundance, providing an approximate readout of transcript levels under the experimental conditions. The students observed that fluorescence intensity for the housekeeping genes remained constant across the three experimental conditions (control, stress, and stress + drug), whereas the test gene associated with oxidative stress pathways exhibited condition-dependent fluctuations, becoming activated or repressed in accordance with its cellular role.

Notable differences emerged among the results obtained by different students, reflecting the influence of their individual experimental decisions and methodological approaches. Consequently, they were required to critically assess the significance and relevance of their findings in relation to their self-designed protocols. Students were able to observe experiments that yielded no detectable results, which they associated with potential errors in cell culture, dose calculation, RNA extraction and handling, preparation of the RT-PCR and PCR mixes, reagent selection, and/or primer design. Those who obtained positive results were able to verify whether the selected HK gene was indeed expressed uniformly across conditions, thereby confirming proper data normalization and the appropriateness of their HK gene choice.

Using a correctly validated HK gene, students observed different expression patterns for the oxidative-stress-related test genes they selected. These included: basal expression level with a slight increase under oxidative stress and reversal upon drug treatment (Figure 2A); basal expression level unaffected by either stress or drug exposure (Figure 2B); basal expression with stress-induced overexpression and only a mild reduction following drug treatment, suggesting limited or no drug efficacy (Figure 2C); and basal or undetectable expression in control cells, strong induction under oxidative stress, and a decrease upon drug treatment (Figure 2D).

### 3.2 Pre–post changes in perceived confidence and familiarity

**FIGURE 3:**
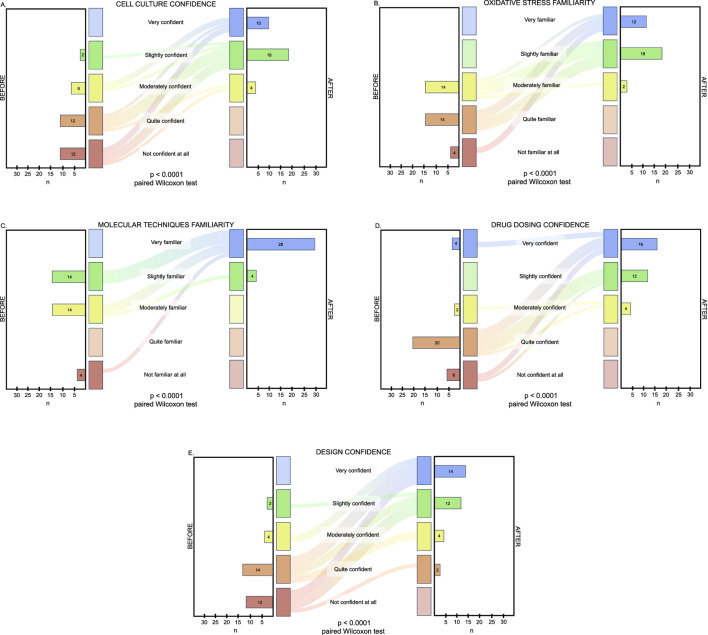
Pre–post changes in students’ perceived confidence and familiarity after the integrated learning experience. Sankey diagrams showing individual changes in students’ responses before and after the intervention for five competency-related items: **(A)** Cell culture confidence, including seeding, propagation, maintenance and sterility procedures; **(B)** Oxidative stress familiarity, referring to genes and cellular mechanisms involved in oxidative stress; **(C)** Molecular techniques familiarity, including RNA extraction, cDNA synthesis and PCR; **(D)** Drug dosing confidence, referring to dose calculation and treatment application in cell cultures; and **(E)** Experimental design confidence, including the design of transcript analysis experiments, selection of genes of interest, experimental conditions, controls and pharmacological treatments. For confidence-based items, response categories ranged from “not confident at all” to “very confident”; for familiarity-based items, categories ranged from “not familiar at all” to “very familiar”. Higher scores indicate greater perceived confidence or familiarity.

A total of 32 valid paired pre–post responses were analyzed for the five items assessing students’ perceived confidence or familiarity before and after the curriculum-integrated learning experience. Students showed significant increases in all five pre–post items after completing the intervention (Figure 3; Table 2). Median scores increased for cell culture confidence from 2 (IQR: 1–2.75) to 4 (IQR: 4–5), oxidative stress familiarity from 2 (IQR: 2–3) to 4 (IQR: 4–5), molecular techniques familiarity from 2 (IQR: 2–2) to 4.5 (IQR: 4–5), drug dosing confidence from 3 (IQR: 3–4) to 5 (IQR: 5–5), and experimental design confidence from 2 (IQR: 1–2) to 4 (IQR: 4–5). All changes were statistically significant using the Wilcoxon matched-pairs signed-rank test (*p* < 0.0001 for all comparisons).

**TABLE 2:** Pre–post changes in students’ perceived confidence and familiarity.

Item	Before, median (IQR)	After, median (IQR)	Improved, n (%)	Unchanged, n (%)	Decreased, n (%)	*p* value
A. Cell culture confidence	2 (1–2.75)	4 (4–5)	30 (93.8%)	2 (6.2%)	0 (0%)	<0.0001
B. Oxidative stress familiarity	2 (2–3)	4 (4–5)	30 (93.8%)	2 (6.2%)	0 (0%)	<0.0001
C. Molecular techniques familiarity	2 (2–2)	4.5 (4–5)	26 (81.3%)	6 (18.7%)	0 (0%)	<0.0001
D. Drug dosing confidence	3 (3–4)	5 (5–5)	32 (100%)	0 (0%)	0 (0%)	<0.0001
E. Experimental design confidence	2 (1–2)	4 (4–5)	30 (93.8%)	2 (6.2%)	0 (0%)	<0.0001

Pre–post comparisons were performed using the Wilcoxon matched-pairs signed-rank test. Responses were rated on a 5-point Likert scale, with higher scores indicating greater perceived confidence or familiarity depending on the item. IQR, interquartile range.

Individual paired transitions showed that most students improved in each item, with no decreases observed among non-tied responses. The proportion of students showing improvement was 93.8% for cell culture confidence, 93.8% for oxidative stress familiarity, 81.3% for molecular techniques familiarity, 100% for drug dosing confidence and 93.8% for experimental design confidence. The Sankey diagrams illustrate a clear shift from lower pre-intervention response categories towards higher post-intervention categories, particularly “quite confident/familiar” and “very confident/familiar” after the intervention.

Overall, these results suggest that the integrated learning experience was associated with broad perceived gains across the cellular, pharmacological and molecular components of the experimental workflow.

### 3.3 Post-intervention perceptions of the integrated learning experience

**FIGURE 4:**
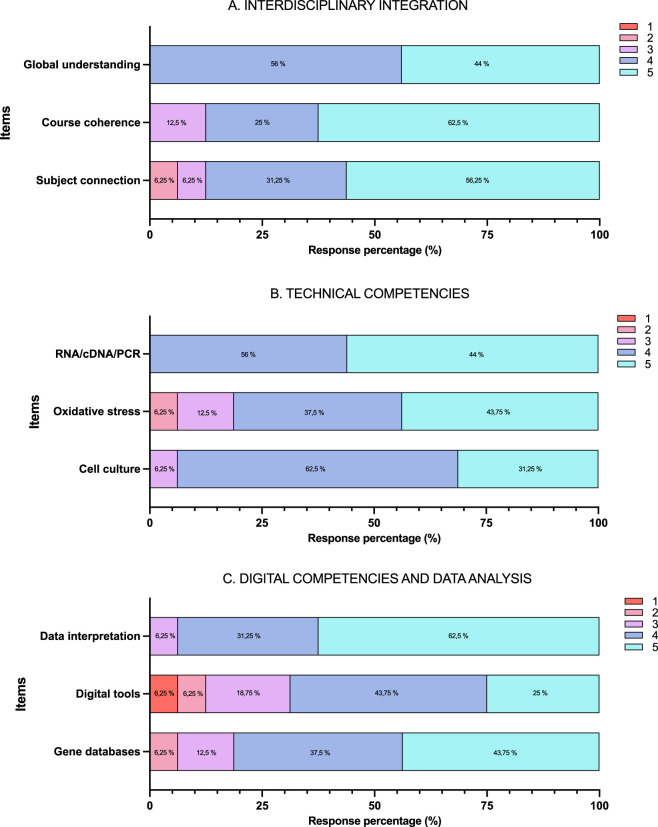
Students’ post-intervention perceptions of the curriculum-integrated learning experience. Stacked horizontal bar plots showing the distribution of students’ responses to post-intervention Likert-scale items after completion of the integrated activity. **(A)** Interdisciplinary integration, including perceived subject connection, course coherence and global understanding of the experimental workflow. **(B)** Technical competencies, including perceived improvement in cell culture, oxidative stress analysis and RNA/cDNA/PCR techniques. **(C)** Digital competencies and data analysis, including perceived usefulness of gene databases, digital tools and data interpretation. Responses were rated on a 5-point Likert scale, with higher scores indicating stronger agreement.

Post-intervention Likert-scale responses showed a generally positive perception of the curriculum-integrated learning experience across the evaluated domains (Figure 4). In the interdisciplinary integration domain, responses were predominantly concentrated in the highest agreement categories, indicating that students perceived a clear connection between cell culture, pharmacology and functional genomics/transcriptomics. Students also reported that the activity helped them understand the coherence between the participating subjects and provided a global view of the experimental workflow.

Positive perceptions were also observed for technical competencies. Students reported high levels of agreement regarding the usefulness of the activity for reinforcing cell culture-related skills, understanding oxidative stress induction and evaluation, and improving their preparedness for RNA extraction, cDNA synthesis and PCR-based analysis. These findings support the role of the intervention as a practical, technique-oriented learning experience in which students could connect cellular handling, pharmacological treatment and molecular readouts.

Regarding digital competencies and data analysis, students positively valued the contribution of the activity to gene database exploration, use of digital tools and interpretation of experimental results. This suggests that the integrated workflow also supported data-related competencies beyond laboratory execution, particularly by linking gene selection, primer design and interpretation of molecular findings within the same experimental context.

Exploratory internal consistency analysis showed Cronbach’s α values of 0.682 for interdisciplinary integration, 0.609 for technical competencies, 0.574 for digital competencies and data analysis, and 0.662 for research and professional development. These values were interpreted as preliminary indicators supporting the descriptive use of the post-intervention domains, considering the purpose-designed nature of the questionnaire and the limited number of items per domain.

**FIGURE 5:**
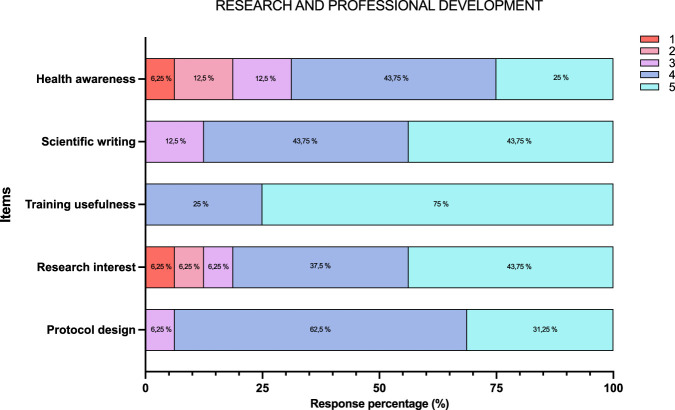
Students’ perceptions of research and professional development after the integrated learning experience. Stacked horizontal bar plot showing students’ post-intervention responses to items related to protocol design, interest in biotechnology research, perceived usefulness for professional training, scientific writing and awareness of the health relevance of oxidative stress. Responses were rated on a 5-point Likert scale, with higher scores indicating stronger agreement.

Students also reported favorable perceptions in the research and professional development domain (Figure 5). Responses indicated that the activity contributed to protocol design, interest in biotechnology research, perceived usefulness for professional training and scientific writing. Students also recognized the relevance of oxidative stress for health-related contexts, suggesting that the intervention helped connect experimental work with broader biomedical and professional implications.

Overall, post-intervention responses indicate that students perceived the activity as coherent, useful and applicable to their training as biotechnology students, with positive evaluations across interdisciplinary, technical, digital, analytical and research-oriented dimensions.

### 3.4 Overall satisfaction and qualitative feedback

Overall satisfaction with the project was high (Table 3). The mean satisfaction score was 8.50 ± 1.10 out of 10, with a median of 8.5 (IQR: 8–9; range: 6–10). Most students rated the activity positively, with 28 of 32 participants (87.5%) giving a score of 8 or higher, and 16 students (50.0%) giving a score of 9 or 10.

**TABLE 3:** Overall student satisfaction with the curriculum-integrated learning experience.

Satisfaction indicator	Total sample (n = 32)
Mean ± SD	8.50 ± 1.10
Median (IQR)	8.5 (8–9)
Range	6–10
Score ≥8, n (%)	28 (87.5%)
Score ≥9, n (%)	16 (50.0%)

Overall satisfaction was assessed using a 1–10 scale, with higher scores indicating greater satisfaction with the project. SD, standard deviation; IQR, interquartile range.

Open-ended responses were grouped into recurrent thematic categories (Table 4). The most frequent positive theme was interdisciplinary integration and global understanding of the experimental workflow, with students emphasizing the value of connecting cell culture, pharmacology and genomics/transcriptomics within a single project. Students also highlighted the acquisition of practical and research-related skills. Suggested improvements were mainly organizational, particularly regarding scheduling, workload distribution, the possibility of submitting a single integrated report, laboratory pairing and clearer practical guidance. Overall, qualitative feedback supported the perceived educational value of the intervention while identifying specific aspects for future refinement.

**TABLE 4:** Summary of open-ended student feedback. Open-ended responses were reviewed and grouped into recurrent thematic categories. Counts refer to coded comment fragments rather than mutually exclusive student responses; therefore, a single response could contribute to more than one category. Representative examples were translated and lightly edited for clarity while preserving the original meaning.

Feedback type	Thematic category	n	Main content included	Representative example
Positive	Interdisciplinary integration and global understanding	11	Comments highlighting the connection between cell culture, pharmacology and genomics/transcriptomics, and the value of understanding the experimental workflow as a whole	“It helped me connect the three subjects and understand the whole process”
Positive	Practical and research-related skill acquisition	6	Comments referring to learning or reinforcing laboratory techniques, transcript analysis, protocol design, PCR/electrophoresis and practical procedures	“Learning how to culture cells and perform PCR and electrophoresis was very useful”
Area for improvement	Scheduling, workload and integrated assessment	5	Comments suggesting earlier scheduling of practical sessions, better workload distribution and preparation of a single integrated report instead of separate reports	“It would be better to prepare one single report integrating all the subjects”
Area for improvement	Laboratory pairing, autonomy and fairness	5	Comments suggesting more autonomy in practical work, flexibility in laboratory pairing or concerns about random pair allocation and unequal workload	“Allowing students to choose laboratory partners could improve the work process”
Area for improvement	Clarity of instructions and interdisciplinary connection	4	Comments suggesting clearer explanation of the relationship between subjects and better organization of laboratory guides or protocols	“The connection between the subjects could be explained more clearly”
Other/Neutral	Minor suggestions or no changes suggested	5	Included one isolated suggestion to increase the number or complexity of practical sessions and two comments indicating that no changes were needed	“No changes; the activity was very complete”

The predefined feasibility and acceptability criteria were met for overall satisfaction, perceived improvement and qualitative feedback. Overall, 28 of 32 students (87.5%) rated the activity with a score of 8 or higher, exceeding the predefined threshold of 80% overall satisfaction. In addition, the proportion of students showing improvement in the pre–post items ranged from 81.3% to 100%, exceeding the predefined threshold of 70% perceived improvement in all five evaluated items.

Qualitative feedback also supported the educational value of the intervention, particularly regarding interdisciplinary integration and global understanding of the experimental workflow. The most frequent positive theme was the perceived connection between cell culture, pharmacology and functional genomics/transcriptomics, together with the value of understanding the project as a coherent research-oriented process.

## 4 Discussion

### 4.1 Principal findings

The main finding of this study is that a curriculum-integrated learning experience linking experimental pharmacology, cell culture and Nrf2-related gene expression analysis was associated with consistent perceived learning gains and high student satisfaction in third-year Biotechnology students. Importantly, the improvements were not restricted to a single technical domain, but involved confidence in cell culture, familiarity with oxidative stress mechanisms and molecular techniques, confidence in drug dosing, and experimental design. This supports the educational value of presenting pharmacological modulation, cellular models and molecular readouts as connected components of the same biotechnology workflow rather than as isolated subject-specific tasks. Crucially, this comprehensive view was reinforced by the student-led nature of the cell culture component, in which students initiated the practical workflow from the early handling of their own A549 cultures and maintained them throughout the project, thereby mimicking a real-world research process within an undergraduate teaching context. In this sense, the intervention is aligned with contemporary approaches in life science education that emphasize authentic, research-oriented and competency-based learning experiences combining experimental work, gene-centered analysis, data interpretation and scientific communication (Yang and Korsnack, 2024; Plaisier et al., 2024; Reed et al., 2025).

A relevant aspect of this experience is that experimental pharmacology functioned as a pedagogical bridge between cellular and molecular learning, without displacing the contribution of the other subjects. The use of sulforaphane as an Nrf2-activating compound and tBHP as a pro-oxidant stimulus allowed students to work with a biologically meaningful question in which treatment design, cellular response and gene expression analysis were conceptually linked. This type of contextualized pharmacological learning is consistent with recent calls to move from content-heavy and discipline-isolated approaches towards learner-centered and competency-oriented pharmacology education (Xiao et al., 2023; Fasinu and Wilborn, 2024). At the same time, the positive responses regarding interdisciplinary integration and global understanding suggest that students valued the activity primarily as an integrated biotechnology experience, in which pharmacology, cell culture and functional genomics/transcriptomics contributed complementary parts of a coherent research-oriented process.

### 4.2 Curriculum integration as a strategy for biotechnology education

The positive perception of interdisciplinary integration observed in this study supports the value of designing biotechnology practical training around a shared experimental problem rather than around isolated laboratory sessions. In this experience, students did not approach cell culture, pharmacological treatment and gene expression analysis as independent technical blocks, but as sequential and interdependent stages of the same research-oriented workflow. This is relevant because biotechnology practice requires students to move across disciplinary boundaries, connecting cellular models, treatment design, molecular mechanisms, digital tools and data interpretation. Although integrated curricula have traditionally been discussed in health professions education (Harden, 2000; Brauer and Ferguson, 2015), recent work continues to emphasize the need to evaluate and operationalize curriculum integration as a measurable educational construct rather than as a simple administrative alignment of subjects (Allouch et al., 2024). In our intervention, the integration was operationalized through a common biological question, shared experimental conditions and a final scientific report requiring students to synthesize the contributions of the three participating subjects.

This structure may explain why students particularly valued subject connection, course coherence and global understanding of the experimental workflow. Rather than learning techniques as disconnected procedures, students were exposed to a continuum in which experimental pharmacology provided the treatment rationale, cell culture provided the biological system, and functional genomics/transcriptomics provided the molecular readout. This approach is consistent with recent undergraduate genomics frameworks, which highlight the importance of helping students connect molecular information with biological function, experimental design and interpretation (Reed et al., 2025). It also aligns with research-oriented curriculum models in molecular life sciences, where integration is strengthened when students participate in authentic tasks such as literature searching, gene selection, primer design, experimental execution and scientific communication (Yang and Korsnack, 2024). Therefore, the educational value of the intervention lies not only in the acquisition of specific technical skills, but in helping students understand how those skills interact to generate biological evidence.

### 4.3 Experimental pharmacology as a bridge between cellular and molecular learning

A distinctive feature of this intervention is that experimental pharmacology acted as the functional link between the cellular and molecular components of the learning experience. The pharmacological stage was not limited to dose calculation or treatment application, but provided the experimental rationale that connected the A549 cellular model with the downstream analysis of Nrf2-related gene expression. By using sulforaphane as an Nrf2-activating compound and tBHP as a pro-oxidant stimulus, students were exposed to a biologically meaningful sequence in which a pharmacological intervention could be interpreted through cellular response and molecular readouts. This is particularly relevant in biotechnology education, where students are expected to understand biological modulation not only as a theoretical mechanism, but also as an experimentally testable process.

Recent literature in pharmacology education supports the need to move beyond content-heavy teaching towards approaches that emphasize core concepts, applied reasoning and competency development. Contemporary reviews describe a shift from lecture-based and discipline-centred pharmacology teaching towards active, learner-centred and competence-based models (Fasinu and Wilborn, 2024; Crowley et al., 2025). Similarly, international efforts to define concept-based pharmacology curricula highlight the importance of helping students understand transferable principles such as drug action, concentration–response relationships, variability, and the interpretation of pharmacological effects (Guilding et al., 2025). In our intervention, these principles were not taught as abstract concepts, but were embedded into an experimental workflow in which students calculated and applied treatments, considered experimental controls and interpreted the possible biological consequences of Nrf2 pathway activation.

The strong improvement observed in drug dosing confidence is therefore educationally relevant, but it should be interpreted within the broader integrated design of the activity. Students did not simply learn how to prepare a compound or apply a stimulus; they were required to understand why those conditions were included and how they contributed to the interpretation of gene expression results. This aligns with recent evidence suggesting that problem-based and integrated learning environments may support pharmacology learning by encouraging students to connect mechanisms, context and application (Nicolaou et al., 2024; Yang et al., 2024). It is also consistent with the wider movement towards competency-based education in biomedical and pharmaceutical sciences, where the emphasis is placed on applying knowledge, skills and judgement to authentic or professionally relevant tasks (ElKhalifa et al., 2024).

Importantly, the pharmacological component did not displace the contribution of cell culture or functional genomics/transcriptomics. Rather, it gave purpose to both: cell culture provided the biological system in which the intervention could be applied, and gene expression analysis provided the molecular readout through which the intervention could be interpreted. This structure may help students understand pharmacology as an experimental and translational discipline that connects chemical or drug-based modulation with measurable biological outcomes. In the context of a Biotechnology degree, this framing is especially valuable because it situates pharmacological reasoning within the broader skillset required for biomedical research, including cellular experimentation, molecular analysis, data interpretation and scientific communication.

### 4.4 Research-oriented, technical and digital competency development

Beyond perceived gains in specific techniques, this intervention exposed students to a research-oriented workflow in which experimental execution, digital tool use and scientific communication were connected to the same biological question. Students were required to search for genes related to oxidative stress and Nrf2 signaling, retrieve sequence information from biological databases, design primers, perform PCR-based gene expression analysis and integrate their findings into a scientific report. This sequence is educationally relevant because it resembles the structure of authentic research practice more closely than isolated laboratory exercises, while remaining feasible within an undergraduate teaching context.

The digital component of the activity was particularly important. Modern biotechnology training increasingly requires students to move between wet-lab procedures and digital resources, including sequence databases, gene annotation tools, primer design software and data interpretation workflows. Recent genomics and bioinformatics education frameworks emphasize that undergraduate students should understand how molecular data are generated, organized and interpreted, rather than learning computational tools as disconnected technical resources (Reed et al., 2025; Yang and Korsnack, 2024). In this study, the use of databases and primer design was not presented as an independent bioinformatics task, but as a necessary step for answering the experimental question generated by the pharmacological and cellular components of the project.

This design also aligns with recent CURE-informed approaches in which students participate in scientific practices such as asking questions, selecting targets, generating data, interpreting evidence and communicating results (Plaisier et al., 2024; Zhang et al., 2025). A key element of this alignment was the autonomy granted to students in managing their own A549 cultures from the initial stages of the project, following an authentic research timeline and making decisions related to cell culture progression, treatment application and sample processing. Although the intervention was not designed as a full-scale discovery-based research project, it incorporated several research-like elements that are valuable for biotechnology education: decision-making, methodological troubleshooting, interpretation of imperfect experimental outputs and scientific writing. Therefore, the activity may have contributed not only to students’ confidence in individual techniques, but also to their understanding of how technical, digital and analytical competencies interact in a coherent experimental workflow.

### 4.5 Student feedback and implementation refinements

The qualitative feedback provided complementary insight into how students experienced the curriculum-integrated activity. The most frequent positive comments referred to interdisciplinary integration and global understanding of the experimental workflow, suggesting that students valued the opportunity to connect experimental pharmacology, cell culture and functional genomics/transcriptomics within a single learning sequence. This reinforces the idea that the perceived value of the intervention was not limited to acquiring individual technical skills, but also involved understanding how different procedures contribute to a shared biological question. Student perceptions and satisfaction are particularly informative in active and laboratory-based learning experiences, where the success of the activity depends not only on the scientific design, but also on how students experience the sequence, workload and coherence of the learning process (Bradley et al., 2023).

At the same time, the open-ended responses identified specific aspects that should be refined in future editions of the activity. Most suggestions were organizational rather than conceptual, including earlier scheduling of practical sessions, clearer practical guides, greater transparency in how the three subjects were connected, and the possibility of preparing a single integrated report instead of separate subject-specific outputs. These comments are relevant because integrated and research-oriented learning experiences require careful coordination, explicit communication of expectations and adequate scaffolding throughout the process. Previous analyses of CUREs and undergraduate laboratory education have emphasized that the value of these experiences depends on the intentional development of scientific practices, rather than on the mere inclusion of practical or research-like activities (Buchanan and Fisher, 2022; Bapu Ramesh et al., 2025).

For future editions of the activity, the intervention could therefore be strengthened by introducing a shared initial briefing explaining the complete workflow, providing a common rubric for the final scientific report, and aligning assessment criteria across the three participating subjects. A single integrated report may also reinforce the central educational message of the project by requiring students to synthesize treatment design, cell culture procedures, molecular analysis and data interpretation in one coherent document. In addition, allowing some flexibility in laboratory pairing, while maintaining fair workload distribution, may improve students’ perception of autonomy and collaboration. These refinements would preserve the research-oriented nature of the activity while addressing the main organizational issues identified by students.

### 4.6 Limitations and future directions

This study has some limitations that should be considered when interpreting the findings. First, the intervention was implemented in a single cohort of third-year Biotechnology students at one institution, which limits the generalizability of the results to other degrees, academic years or educational contexts. Second, the study used a one-group pre–post design without a control group or comparison with a non-integrated teaching approach; therefore, the observed improvements cannot be attributed exclusively to the intervention. Other factors, such as progressive exposure to course content, maturation during the semester or response-shift effects, may have contributed to the reported changes. Accordingly, the findings should be interpreted as perceived learning gains associated with the integrated learning experience. Third, the main outcomes were based on self-reported confidence, familiarity, satisfaction and perceptions, which may be influenced by response bias, social desirability or students’ positive engagement with the activity. In addition, objective learning outcomes, such as experimental report scores, experimental operation scores or standardized assessment of the accuracy of gene expression interpretation, were not systematically analyzed as primary endpoints. Therefore, the present findings should be interpreted as evidence of perceived learning and educational acceptability rather than as direct evidence of objective competency acquisition. Finally, no formal evaluation of teaching staff satisfaction, workload or coordination demands was conducted, and instructor perceptions were therefore not analyzed as study outcomes.

Future research should replicate the intervention in larger and successive cohorts, include objective measures of learning and performance, and compare integrated learning experiences with non-integrated or traditional teaching approaches. Such objective measures could include assessment of experimental reports, laboratory performance and accuracy of data interpretation. Future studies should also evaluate whether this type of intervention improves long-term retention of experimental, analytical and research-related competencies. Future implementations should also incorporate structured instructor feedback on feasibility, workload, coordination demands and satisfaction with the integrated format, particularly if the activity is replicated in larger cohorts or implemented by broader teaching teams. In addition, student focus groups may complement questionnaire-based evaluation by providing more nuanced information on the organization, perceived value and sustainability of the integrated learning experience (Bourne and Winstone, 2021).

## 5 Conclusion

This study shows that a curriculum-integrated learning experience linking experimental pharmacology, cell culture and Nrf2-related gene expression analysis was associated with positive perceived learning gains, high satisfaction and favorable student perceptions in undergraduate Biotechnology education. By connecting treatment design, cellular models, molecular analysis, data interpretation and scientific writing within a shared experimental workflow, the intervention helped students understand the relationship between different disciplinary components of biomedical research. Although further studies including objective learning measures, larger cohorts and comparative designs are needed, this experience provides a transferable model for integrating experiential, research-oriented and competency-based learning in biotechnology degrees.

## Data Availability

The raw data supporting the conclusions of this article will be made available by the authors, without undue reservation.
