# Supplementary material for: A curriculum-integrated learning experience linking experimental pharmacology, cell culture and Nrf2-related gene expression analysis in undergraduate biotechnology education
Source: Front Pharmacol. 2026 Jun 29;17:1889438. doi: 10.3389/fphar.2026.1889438 (PMC13357193; doi:10.3389/fphar.2026.1889438)
Supplement: Supplementary file 1 [file DataSheet1.DOCX]

**Supplementary Material 1. Student questionnaire**

***English Version.***

Unless otherwise stated, closed-ended questions were answered using a **5-point Likert scale**, where higher scores indicated greater confidence, familiarity or agreement with the statement. Overall satisfaction was assessed using a **1–10 scale**.

**Informed consent**

1. I consent to the anonymous use of my responses for teaching and educational research purposes.
   - Yes
   - No
2. I consent to the anonymous use of the results obtained in this project for teaching and educational research purposes.
   - Yes
   - No

**Pre- and post-intervention perceived learning**

1. Before participating in this project, what was your level of confidence in working with cell cultures, including seeding, propagation/growth, maintenance and sterility in a biosafety cabinet?
2. After participating in this activity, what is your level of confidence in working with cell cultures, including seeding, propagation/growth, maintenance and sterility in a biosafety cabinet?
3. Before participating in this activity, what was your level of familiarity with the genes and cellular mechanisms involved in oxidative stress?
4. After participating in this activity, what is your level of familiarity with the genes and cellular mechanisms involved in oxidative stress?
5. Before participating in this activity, what was your level of familiarity with RNA extraction, cDNA synthesis and PCR techniques?
6. After participating in this activity, what is your level of familiarity with RNA extraction, cDNA synthesis and PCR techniques?
7. Before participating in this activity, what was your level of confidence in calculating drug doses and applying them to cell cultures?
8. After participating in this activity, what is your level of confidence in calculating drug doses and applying them to cell cultures?
9. Before participating in this activity, what was your level of confidence in designing a transcript analysis experiment in cell cultures, including the search for genes of interest, experimental conditions, controls and drugs to be explored?
10. After participating in this activity, what is your level of confidence in designing a transcript analysis experiment in cell cultures, including the search for genes of interest, experimental conditions, controls and drugs to be explored?

**A. Interdisciplinary integration**

1. The activity helped me understand the relationship between cell culture, pharmacology and transcriptomics.
2. I perceived coherence between the subjects involved in the project.
3. The project allowed me to understand the complete experimental process in a global way.

**B. Technical competencies**

1. I feel more confident working with cell cultures.
2. I have a better understanding of how oxidative stress is induced and evaluated.
3. I feel better prepared to perform RNA extraction, cDNA synthesis and PCR.

**C. Digital competencies and data analysis**

1. The bioinformatics seminars helped me find, study and understand the genes involved in oxidative stress processes.
2. I feel better prepared to use digital tools and data analysis resources in research, including databases, software such as ApE and MEGA, and statistical analysis.
3. The activity improved my ability to interpret experimental results.

**D. Critical thinking and social awareness**

1. This experience improved my ability to propose experimental protocols.
2. Participating in this project increased my interest in biotechnology research.
3. I consider this experience useful for my training as a biotechnologist.
4. Writing the scientific article helped me integrate and consolidate my learning.
5. This activity increased my awareness of the impact of oxidative stress on global health.

**Open-ended questions**

1. What was the most useful or meaningful aspect of this experience?
2. What aspects of the project would you improve?
3. Other comments. If you have no additional comments, please write “Not applicable”.

**Overall satisfaction**

1. On a scale from 1 to 10, what is your overall level of satisfaction with the project?

***Spanish Version*.**

**Encuesta de recogida de datos del alumnado**

Todas las preguntas cerradas, salvo indicación contraria, se respondieron mediante una escala tipo Likert de **1 a 5**, donde los valores más altos indicaban mayor nivel de confianza, familiaridad o acuerdo con la afirmación. La satisfacción global se evaluó mediante una escala de **1 a 10**.

**Consentimiento informado**

1. Consiento la utilización anónima de mis respuestas para fines docentes y de investigación educativa.
   - Sí
   - No
2. Consiento la utilización anónima de los resultados obtenidos en este proyecto para fines docentes y de investigación educativa.
   - Sí
   - No

**Percepción previa y posterior de aprendizaje**

1. Antes de realizar este proyecto, ¿cuál es tu nivel de confianza para trabajar con cultivos celulares, incluyendo siembra, propagación/crecimiento, mantenimiento y esterilidad en campana?
2. Después de realizar esta actividad, ¿cuál es tu nivel de confianza para trabajar con cultivos celulares, incluyendo siembra, propagación/crecimiento, mantenimiento y esterilidad en campana?
3. Antes de realizar esta actividad, ¿qué nivel de familiaridad tienes con los genes y mecanismos celulares que participan en el estrés oxidativo?
4. Después de realizar esta actividad, ¿qué nivel de familiaridad tienes con los genes y mecanismos celulares que participan en el estrés oxidativo?
5. Antes de realizar esta actividad, ¿qué nivel de familiaridad tienes con las técnicas de extracción de RNA, síntesis de cDNA y PCR?
6. Después de realizar esta actividad, ¿qué nivel de familiaridad tienes con las técnicas de extracción de RNA, síntesis de cDNA y PCR?
7. Antes de realizar esta actividad, ¿qué nivel de confianza tienes para calcular dosis de fármacos e inocularlos en cultivos celulares?
8. Después de realizar esta actividad, ¿qué nivel de confianza tienes para calcular dosis de fármacos e inocularlos en cultivos celulares?
9. Antes de realizar esta actividad, ¿qué nivel de confianza tienes para diseñar un experimento de análisis de transcritos en cultivos celulares, incluyendo búsqueda de genes de interés, condiciones experimentales, controles y fármacos a explorar?
10. Después de realizar esta actividad, ¿qué nivel de confianza tienes para diseñar un experimento de análisis de transcritos en cultivos celulares, incluyendo búsqueda de genes de interés, condiciones experimentales, controles y fármacos a explorar?

**A. Integración interdisciplinaria**

1. La actividad me ayudó a comprender la relación entre cultivos celulares, farmacología y transcriptómica.
2. Percibo coherencia entre las asignaturas implicadas en el proyecto.
3. El proyecto me permitió entender todo el proceso experimental de forma global.

**B. Competencias técnicas**

1. Me siento más seguro/a trabajando con cultivos celulares.
2. Comprendo mejor cómo se induce y evalúa el estrés oxidativo.
3. Me siento más preparado/a para realizar extracción de RNA, síntesis de cDNA y PCR.

**C. Competencias digitales y análisis de datos**

1. Los seminarios de bioinformática me ayudaron a encontrar, estudiar y entender los genes implicados en los procesos de estrés oxidativo.
2. Me siento más preparado/a para utilizar herramientas digitales y de análisis de datos en investigación, incluyendo bases de datos, programas tipo ApE, MEGA y análisis estadístico.
3. La actividad mejoró mi capacidad para interpretar resultados experimentales.

**D. Pensamiento crítico y conciencia social**

1. Esta experiencia mejoró mi capacidad para proponer protocolos experimentales.
2. Participar en este proyecto aumentó mi interés por la investigación biotecnológica.
3. Considero que esta experiencia es útil para mi formación como biotecnólogo/a.
4. La elaboración del artículo científico me ha ayudado a integrar y consolidar el aprendizaje.
5. Esta actividad incrementó mi conciencia sobre el impacto del estrés oxidativo en la salud global.

**Preguntas abiertas**

1. ¿Qué ha sido lo más útil o significativo de esta experiencia?
2. ¿Qué aspectos mejorarías del proyecto?
3. Otros comentarios. Si no tienes ningún comentario que añadir, indica “No aplica”.

**Satisfacción global**

1. En una escala del 1 al 10, ¿cuál es tu nivel de satisfacción general con el proyecto?
